# Supplementary material for: Optimal use of statistical methods to validate reference gene stability in longitudinal studies
Source: PLoS One. 2019 Jul 23;14(7):e0219440. doi: 10.1371/journal.pone.0219440 (PMC6650036; doi:10.1371/journal.pone.0219440)
Supplement: S1 Table — The Cq values of all samples are linearized. The CV is then calculated as the ratio of the SD to the mean of the linearized Cq values. It is expressed as a percentage. A lower CV value would mean lower variation across samples and therefore higher stability. (DOCX) [file pone.0219440.s001.docx]

**Supporting Information**

**S1 Table. CV analysis example.** The Cq values of all samples are linearized. The CV is then calculated as the ratio of the SD to the mean of the linearized Cq values. It is expressed as a percentage. A lower CV value would mean lower variation across samples and therefore higher stability.

|  | **CV analysis ACTB** |  |
| --- | --- | --- |
| **Sample** | **ACTB Cq** | **2^-Cq^** |
| **P5_1** | 16.73 | 9.2E-06 |
| **P5_2** | 16.43 | 1.1E-05 |
| **P5_3** | 16.67 | 9.6E-06 |
| **P5_4** | 16.63 | 9.8E-06 |
| **P5_5** | 17.68 | 4.8E-06 |
| **P10_1** | 16.22 | 1.3E-05 |
| **P10_2** | 16.81 | 8.7E-06 |
| **P10_3** | 16.69 | 9.5E-06 |
| **P10_4** | 15.97 | 1.6E-05 |
| **P10_5** | 16.60 | 1.0E-05 |
| **P10_6** | 16.77 | 9.0E-06 |
| **P15_1** | 16.76 | 9.0E-06 |
| **P15_2** | 16.75 | 9.1E-06 |
| **P15_3** | 16.94 | 8.0E-06 |
| **P15_4** | 16.99 | 7.7E-06 |
| **P15_5** | 16.76 | 9.0E-06 |
| **P15_6** | 16.91 | 8.1E-06 |
| **P23_1** | 17.69 | 4.7E-06 |
| **P23_2** | 17.32 | 6.1E-06 |
| **P23_3** | 17.39 | 5.8E-06 |
| **P23_4** | 17.58 | 5.1E-06 |
| **P23_5** | 17.72 | 4.6E-06 |
| **P23_6** | 17.24 | 6.4E-06 |
|  |  |  |
|  | **Mean 2^-Cq^** | **8.4E-06** |
|  | **SD 2^-Cq^** | **2.7E-06** |
|  | **CV (SD/Mean) x100** | **31.86%** |
