# Supplementary material for: Optimal use of statistical methods to validate reference gene stability in longitudinal studies
Source: PLoS One. 2019 Jul 23;14(7):e0219440. doi: 10.1371/journal.pone.0219440 (PMC6650036; doi:10.1371/journal.pone.0219440)
Supplement: S2 Table — The table shows the stability calculation by the Pairwise ΔCt method for Actb. It is a table of Cq value differences between Actb and all the other genes. Each row represents a sample and each column represents a gene. The difference in Cq values between Actb and the others is first calculated across all samples. Therefore, the first column has 0s (difference in Cq of Actb with itself) Next, the standard deviation of the differences is calculated at the end of each column indicated by SD. The stability value (Average SD) of Actb is then calculated as the arithmetic mean of these standard deviations. (DOCX) [file pone.0219440.s002.docx]

**SUPPORTING INFORMATION**

**S2 Table.** **Pairwise ΔCt analysis example.** The table shows the stability calculation by the Pairwise ΔCt method for *Actb*. It is a table of Cq value differences between *Actb* and all the other genes. Each row represents a sample and each column represents a gene. The difference in Cq values between *Actb* and the others is first calculated across all samples. Therefore, the first column has 0s (difference in Cq of *Actb* with itself) Next, the standard deviation of the differences is calculated at the end of each column indicated by SD. The stability value (Average SD) of *Actb* is then calculated as the arithmetic mean of these standard deviations.

|  | ACTB | HSP60 | GAPDH | SDHA | TBP | MRPL10 | PGK | RPL13A | PPIA | RPS26 |
| --- | --- | --- | --- | --- | --- | --- | --- | --- | --- | --- |
| ACTB vs all | 0.00 | 3.65 | 0.94 | 4.10 | 7.47 | 6.07 | 4.58 | 1.35 | 0.33 | 4.58 |
|  | 0.00 | 3.52 | 0.94 | 4.08 | 7.29 | 6.25 | 4.54 | NA | -0.60 | 4.66 |
|  | 0.00 | 3.82 | 1.01 | 4.18 | 7.76 | 6.21 | 4.72 | 1.24 | 0.42 | 4.50 |
|  | 0.00 | 3.18 | 0.76 | 3.63 | 6.70 | 5.92 | 4.08 | 1.08 | -0.08 | 3.84 |
|  | 0.00 | 3.48 | 0.79 | 3.48 | 6.75 | 5.87 | 4.34 | 1.01 | 0.00 | 4.23 |
|  | 0.00 | 5.44 | 2.05 | 3.67 | 8.50 | 5.45 | 3.53 | 0.59 | -0.05 | NA |
|  | 0.00 | 5.11 | 2.58 | 3.86 | 8.19 | 5.84 | 4.37 | 0.56 | -0.01 | NA |
|  | 0.00 | 5.90 | 2.63 | 4.99 | 9.05 | 6.27 | 4.69 | 1.60 | 1.23 | 4.32 |
|  | 0.00 | 4.86 | 1.96 | 3.65 | 8.44 | 5.54 | 3.85 | 0.82 | 0.11 | 4.17 |
|  | 0.00 | 4.19 | 1.62 | 3.67 | 7.83 | 5.59 | 3.77 | 0.55 | -1.07 | 4.19 |
|  | 0.00 | 4.91 | 1.69 | 3.43 | 8.06 | 5.54 | 3.93 | 0.64 | -0.09 | 4.07 |
|  | 0.00 | 4.41 | 1.35 | 3.55 | 8.90 | 5.75 | 3.93 | 1.34 | 0.32 | 5.00 |
|  | 0.00 | 3.90 | 1.38 | 2.93 | 8.02 | 5.45 | 3.49 | 1.07 | -0.23 | 4.46 |
|  | 0.00 | 4.49 | 1.52 | 3.10 | 8.25 | 5.61 | 3.22 | 0.77 | -0.30 | 4.24 |
|  | 0.00 | 4.11 | 1.56 | 3.01 | 7.89 | 5.68 | 3.53 | 0.95 | NA | 4.60 |
|  | 0.00 | 3.87 | 1.34 | 3.04 | 7.73 | 5.32 | 3.29 | 0.76 | -0.26 | 4.50 |
|  | 0.00 | 4.58 | 1.68 | 3.15 | 8.40 | 5.47 | NA | 0.76 | -0.13 | 4.53 |
|  | 0.00 | 2.91 | -0.84 | 1.76 | 5.87 | 4.68 | 1.90 | 0.94 | -1.23 | 3.84 |
|  | 0.00 | 3.02 | -0.65 | 1.82 | 5.77 | 4.78 | 2.03 | 1.43 | -0.94 | 3.73 |
|  | 0.00 | 2.85 | -0.63 | 1.79 | 5.67 | 4.98 | 2.16 | 1.25 | -0.98 | 4.49 |
|  | 0.00 | 3.03 | -0.79 | 1.70 | 5.83 | 5.18 | 1.90 | 1.25 | -1.04 | 4.18 |
|  | 0.00 | 2.68 | -0.96 | 1.75 | 5.79 | 4.75 | 1.83 | 0.98 | -1.00 | 3.98 |
|  | 0.00 | 2.74 | -0.88 | 1.61 | 5.79 | 5.04 | 2.31 | 0.96 | -0.31 | 4.08 |
| **SD** | **0.000** | **0.913** | **1.143** | **0.957** | **1.123** | **0.472** | **0.996** | **0.299** | **0.601** | **0.315** |
|  |  |  |  |  |  |  |  |  |  |  |
| **Average SD** | **0.758** |  |  |  |  |  |  |  |  |  |
