# Supplementary material for: Optimal use of statistical methods to validate reference gene stability in longitudinal studies
Source: PLoS One. 2019 Jul 23;14(7):e0219440. doi: 10.1371/journal.pone.0219440 (PMC6650036; doi:10.1371/journal.pone.0219440)
Supplement: S3 Table — (DOCX) [file pone.0219440.s003.docx]

**Supporting Information**

**S3 Table. Pearson’s correlation matrix for the cerebellum.**

| **Corr. Matrix** | ACTB | HSP60 | GAPDH | SDHA | TBP | MRPL10 | PGK | RPL13A | PPIA | RPS26 |
| --- | --- | --- | --- | --- | --- | --- | --- | --- | --- | --- |
| ACTB |  |  |  |  |  |  |  |  |  |  |
| HSP60 | -0.141 |  |  |  |  |  |  |  |  |  |
| GAPDH | -0.495 | 0.731 |  |  |  |  |  |  |  |  |
| SDHA | -0.36 | 0.438 | 0.817 |  |  |  |  |  |  |  |
| TBP | -0.44 | 0.802 | 0.937 | 0.735 |  |  |  |  |  |  |
| MRPL10 | 0.572 | -0.021 | 0.06 | 0.431 | 0.037 |  |  |  |  |  |
| PGK | -0.292 | 0.291 | 0.74 | 0.93 | 0.652 | 0.538 |  |  |  |  |
| RPL13A | 0.884 | -0.331 | -0.521 | -0.225 | -0.462 | 0.705 | -0.128 |  |  |  |
| PPIA | 0.393 | 0.267 | 0.095 | 0.212 | 0.175 | 0.546 | 0.307 | 0.544 |  |  |
| RPS26 | 0.802 | 0.095 | -0.217 | -0.13 | -0.084 | 0.615 | -0.098 | 0.791 | 0.405 |  |
|  |  |  |  |  |  |  |  |  |  |  |
|  |  |  |  |  |  |  |  |  |  |  |
|  |  |  |  |  |  |  |  |  |  |  |
| **P Values** | ACTB | HSP60 | GAPDH | SDHA | TBP | MRPL10 | PGK | RPL13A | PPIA | RPS26 |
| ACTB |  |  |  |  |  |  |  |  |  |  |
| HSP60 | 5.23E-01 |  |  |  |  |  |  |  |  |  |
| GAPDH | 1.63E-02 | 7.53E-05 |  |  |  |  |  |  |  |  |
| SDHA | 9.12E-02 | 3.66E-02 | 2.01E-06 |  |  |  |  |  |  |  |
| TBP | 3.56E-02 | 4.28E-06 | 4.89E-11 | 6.53E-05 |  |  |  |  |  |  |
| MRPL10 | 4.33E-03 | 9.24E-01 | 7.86E-01 | 4.01E-02 | 8.67E-01 |  |  |  |  |  |
| PGK | 1.88E-01 | 1.89E-01 | 8.32E-05 | 3.80E-10 | 1.01E-03 | 9.77E-03 |  |  |  |  |
| RPL13A | 4.95E-08 | 1.32E-01 | 1.29E-02 | 3.13E-01 | 3.05E-02 | 2.46E-04 | 5.80E-01 |  |  |  |
| PPIA | 7.04E-02 | 2.30E-01 | 6.73E-01 | 3.43E-01 | 4.36E-01 | 8.54E-03 | 1.75E-01 | 1.09E-02 |  |  |
| RPS26 | 1.24E-05 | 6.81E-01 | 3.46E-01 | 5.76E-01 | 7.16E-01 | 2.98E-03 | 6.81E-01 | 3.25E-05 | 7.65E-02 |  |
