# Supplementary material for: Optimal use of statistical methods to validate reference gene stability in longitudinal studies
Source: PLoS One. 2019 Jul 23;14(7):e0219440. doi: 10.1371/journal.pone.0219440 (PMC6650036; doi:10.1371/journal.pone.0219440)
Supplement: S4 Table — (DOCX) [file pone.0219440.s004.docx]

**Supporting Information**

**S4 Table. Pearson’s correlation matrix for the spinal cord.**

| **Corr Matrix** | ACTB | HSP60 | GAPDH | SDHA | TBP | MRPL10 | PGK | RPL13A | PPIA | RPS26 |
| --- | --- | --- | --- | --- | --- | --- | --- | --- | --- | --- |
| ACTB |  |  |  |  |  |  |  |  |  |  |
| HSP60 | 0.723 |  |  |  |  |  |  |  |  |  |
| GAPDH | 0.719 | 0.945 |  |  |  |  |  |  |  |  |
| SDHA | 0.75 | 0.934 | 0.889 |  |  |  |  |  |  |  |
| TBP | 0.734 | 0.919 | 0.953 | 0.872 |  |  |  |  |  |  |
| MRPL10 | 0.81 | 0.9 | 0.815 | 0.943 | 0.83 |  |  |  |  |  |
| PGK | 0.745 | 0.958 | 0.893 | 0.97 | 0.886 | 0.934 |  |  |  |  |
| RPL13A | 0.709 | 0.864 | 0.814 | 0.959 | 0.835 | 0.934 | 0.952 |  |  |  |
| PPIA | 0.643 | 0.829 | 0.803 | 0.686 | 0.864 | 0.681 | 0.726 | 0.609 |  |  |
| RPS26 | -0.007 | -0.089 | -0.041 | -0.334 | 0.043 | -0.291 | -0.278 | -0.413 | 0.356 |  |
|  |  |  |  |  |  |  |  |  |  |  |
| **P values** | ACTB | HSP60 | GAPDH | SDHA | TBP | MRPL10 | PGK | RPL13A | PPIA | RPS26 |
| ACTB |  |  |  |  |  |  |  |  |  |  |
| HSP60 | 6.48E-05 |  |  |  |  |  |  |  |  |  |
| GAPDH | 7.48E-05 | 3.51E-12 |  |  |  |  |  |  |  |  |
| SDHA | 2.46E-05 | 2.50E-11 | 6.62E-09 |  |  |  |  |  |  |  |
| TBP | 4.41E-05 | 2.42E-10 | 6.57E-13 | 2.80E-08 |  |  |  |  |  |  |
| MRPL10 | 1.62E-06 | 2.24E-09 | 1.25E-06 | 5.38E-12 | 5.33E-07 |  |  |  |  |  |
| PGK | 4.58E-05 | 7.40E-13 | 1.01E-08 | 1.87E-14 | 1.93E-08 | 7.13E-11 |  |  |  |  |
| RPL13A | 1.04E-04 | 5.21E-08 | 1.30E-06 | 1.75E-13 | 3.84E-07 | 2.52E-11 | 2.72E-12 |  |  |  |
| PPIA | 6.98E-04 | 5.57E-07 | 2.31E-06 | 2.17E-04 | 5.53E-08 | 2.48E-04 | 8.66E-05 | 1.57E-03 |  |  |
| RPS26 | 9.74E-01 | 6.81E-01 | 8.49E-01 | 1.11E-01 | 8.43E-01 | 1.68E-01 | 1.99E-01 | 4.48E-02 | 8.81E-02 |  |
